# Can We Prevent, Delay, or Shorten the Course of Dementia?

**DOI:** 10.1371/journal.pmed.0030430

**Published:** 2006-10-31

**Authors:** Willem A Van Gool

## Abstract

Van Gool discusses a new study that found that the prevalence of dementia and severe cognitive impairment in the year before death rises steeply with age.

“You already know how it ends when you are born”, my mother said in sorrow after my father died. And she is not even medically qualified. Should we extend this common wisdom on the realities of old age towards the development of dementia? In other words, is dementia a curse that is inescapably linked to old age?

## A New Study on Dementia before Death

In a new study in *PLoS Medicine*, Carol Brayne and colleagues show that in a large population sample, the prevalence of dementia and severe cognitive impairment in the year before death rises steeply with age [1]. The researchers found that the prevalence of dementia in the year before death is up to 40%–60% in those over 90 years of age. They also found that nearly 80% of those dying over 95 years of age suffered from moderate or more severe cognitive impairment.

The researchers examined whether higher levels of education and higher social class (proxies for a healthy lifestyle, e.g., taking exercise and not smoking) protected people from dementia by the time they died. While they did find such a protective effect, the size of this effect was small: higher social class reduced the risk of dying with dementia by only 2%, and higher levels of education reduced the risk by only 7%.

## Policy and Clinical Implications

These figures are important and sobering for policy makers and for those who believe that dementia is preventable. It is difficult to envisage preventive measures that would effectively counteract the profound effects of increasing age on the risk of dementia. As populations age, the burden of dementia will increase, and societies should be prepared for large numbers of elderly patients with dementia. Wishful thinking on prevention of dementia should not defer from the societal investments that are required for providing high quality care for these patients. That is an important message for policy makers and health-care workers alike.

**Figure 1:**
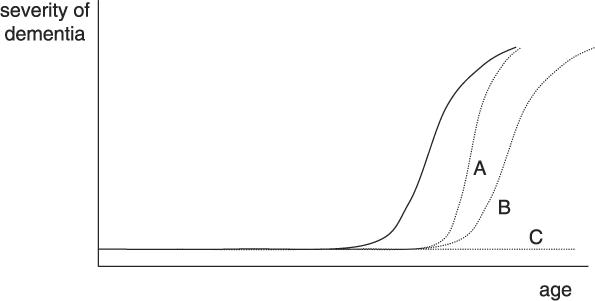
Curves Depicting Severity of Dementia Depending on Age The solid line gives an estimate of a present individual trajectory. Broken lines illustrate the potential benefit of (A) postponing and shortening the episode with dementia, (B) delaying dementia while reaching a higher age, or (C) preventing dementia.

While acknowledging both the methodological rigor of Brayne and colleagues' analysis and the sobering nature of their data, it is important to note that their dataset does allow for slightly more hopeful interpretations. First, the authors' strong emphasis on the large proportion of patients with dementia *at the time of death* must not let us forget about the importance of the age of onset or the duration of dementia. Their figures do not preclude the possibility that substantial gains might be made by delaying the onset of dementia, shortening the period with dementia, or both (Figure 1). Such scenarios, which would not change the total number of patients suffering from dementia in the period before death, could yield enormous benefits in terms of alleviating suffering at younger ages and reducing the burden for families and other informal caregivers.

Second, the observational nature of the present study limits its interpretation in terms of cause and effects, especially with respect to the small effects of potential protective factors. The important role of cardiovascular risk factors for dementia and more specifically for Alzheimer disease has been recognized for almost ten years [2]. This has fuelled the hope that at least some components of dementia may be amenable to preventive measures. Both observational and trial data suggest that treatment of hypertension does indeed protect against dementia, and the same may hold for interventions directed against obesity, smoking, lack of exercise, and glucose intolerance [3–10].

As acknowledged by Brayne and colleagues, stratification according to educational levels and social class—as proxies for the level of cardiovascular prevention—is not very strong. Physical fitness, body weight, smoking behaviour, blood pressure, and glucose intolerance might have been better indicators, but even then observational studies like theirs, which can be biased by confounding factors, may fail to define the potential for vascular prevention of dementia.

## The Need for Randomised Controlled Trials

Only properly designed randomised controlled trials of sufficient power and sufficiently long follow up can convincingly define the window of opportunity for prevention of dementia. Such studies, which will be a major undertaking, can also document the precise effects of risks that may compete with the onset of dementia. Delaying the onset of dementia by, say, six to eight years may unmask other diseases in the elderly such as ischemic heart disease or cancer, which would have gone undetected if the dementia had not been delayed. The effects of these competing risks on survival may eventually determine if delaying the onset of dementia may ultimately result in prevention of dementia (Figure 1).

Individuals who develop dementia at the age of 75 or 85 years may differ in more respects than just the tendency to develop dementia. Therefore, in the absence of randomisation and without an intervention that protects individuals who would have developed dementia at a given age, observational studies can never precisely define the effects of risks that may compete with the onset of dementia.

Direct studies of the beneficial and adverse effects of preventive measures are required irrespective of their target, being either ß-amyloid accumulation in the brain or vascular brain damage. At least one such study is now under way (“Prevention of dementia by intensive vascular care [pre-DIVA]”, ISRCTN #29711771). Because of the long follow up that is required in studies of primary prevention, it will be several years before we will know if the one-way funnel leading towards nursing home placement can be turned into a freeway leading to a life free from dementia at old age.
